# Short-Term Oral Quercetin Supplementation Improves Post-exercise Insulin Sensitivity, Antioxidant Capacity and Enhances Subsequent Cycling Time to Exhaustion in Healthy Adults: A Pilot Study

**DOI:** 10.3389/fnut.2022.875319

**Published:** 2022-04-28

**Authors:** Jung-Piao Tsao, Jeffrey R. Bernard, Hsiu-Chen Hsu, Chin-Lin Hsu, Su-Fen Liao, I-Shiung Cheng

**Affiliations:** ^1^Department of Sports Medicine, China Medical University, Taichung City, Taiwan; ^2^Department of Kinesiology, California State University, Stanislaus, Turlock, CA, United States; ^3^Physical Education Office, Central Taiwan University of Science and Technology, Taichung City, Taiwan; ^4^School of Nutrition, Chung Shan Medical University, Taichung City, Taiwan; ^5^Department of Nutrition, Chung Shan Medical University Hospital, Taichung City, Taiwan; ^6^Department of Physical Medicine and Rehabilitation, Changhua Christian Hospital, Changhua City, Taiwan; ^7^Department of Post-Baccalaureate Medicine, College of Medicine, National Chung Hsing University, Taichung City, Taiwan; ^8^Department of Physical Education, National Taichung University of Education, Taichung City, Taiwan

**Keywords:** exercise, time to exhaustion, ergogenic aids, quercetin, insulin-stimulated glucose uptake

## Abstract

**Aim:**

Quercetin has been reported to have antioxidant and anti-inflammatory properties on health promotion in human studies. The main purpose of this study was to investigate the effect of short-term oral quercetin supplementation on post-exercise whole-body energy metabolism. This study also aimed to determine the effects of supplementation on oxygen stress, inflammation, muscle damage, and high-intensity cycling exercise performance.

**Method:**

Twelve healthy participants, physically active students, were recruited to perform a randomized, single-blind crossover study. All subjects completed 7-days of quercetin (quercetin:1,000 mg per day for 7-days) and placebo supplementation in a randomized order. Supplement/placebo was combined with exercise consisting of 70% V̇O_2max_ cycling for 60-min, followed by 3-h of recovery, then a subsequent single bout of cycling exercise with 75% V̇O_2max_ to exhaustion. Time to exhaustion, indicators of muscle damage, as well as blood and gaseous parameters relating to energy metabolism, oxidative stress, inflammatory response, respectively, were determined.

**Results:**

The results showed that 7-day quercetin supplementation significantly attenuated the post-exercise glucose-induced insulin response, increased total antioxidant capacity (TAC) and superoxidase dismutase (SOD) activities, and mitigated malondialdehyde (MDA) levels during the recovery period (*p* < 0.05). While subsequent 75% V̇O_2max_ cycling performance was significantly improved after quercetin treatment and accompanied by lower responses of interleukin 6 and creatine kinase at 24-h. However, it’s noted that there were no significant responses in glucose, respiratory exchange rate, tumor necrosis factor-α (TNF-α), myoglobin, and high sensitivity C-reactive protein between quercetin and placebo trials.

**Conclusion:**

Our findings concluded that 7-day oral quercetin supplementation enhances high-intensity cycling time to exhaustion, which may be due in part to the increase in whole-body insulin-stimulated glucose uptake and attenuation of exercise-induced oxygen stress and pro-inflammation. Therefore, quercetin may be considered an effective ergogenic aid for enhancing high-intensity cycling performance among young adults.

## Introduction

Attenuating oxidative stress, inflammation, and muscle damage induced by high-intensity exercise is the key factor for improving exercise performance (1). These effects have been shown in both animal and human models. Uchiyama et al. reported that rats had elevated levels of reactive oxygen species (ROS) and malondialdehyde (MDA) following repeated maximal resistance exercise until fatigue (2). This same study also indicated that muscle damage to the plantar flexor was highly associated with creatine kinase (CK) concentrations (2). As for humans, high-intensity exercise such as running up and down hills for 60 min has been shown to increase oxidative stress, inflammation, and muscle damage (3). Yet another study, involving 18 athletes, found that the concentration of thioredoxin (TRX) and myeloperoxidase (MPO) increased after completing a triathlon (1). In addition, an increase was also observed in CK levels in response to high-intensity triathlon exercise (1). Interestingly, 3-week supplementation of lactobacillus Plantarum PS128, an antioxidant supplement, attenuated exercise-induced oxidative stress and muscle damage levels which resulted in improved performance among the triathletes (1). Therefore, Huang’s study suggests that assuaging oxidative stress, inflammation, and muscle damage induced by mid- and high-intensity exercise may be an important physiological factor for enhancing performance.

Quercetin is a type of phytochemical belonging to the flavonoid family, hence it has antioxidant effects and the ability to decrease free radicals in the body (4). Quercetin can be purified from fruits and vegetables including onion, blueberry, and broccoli. Results from animal cell and human experiments show that quercetin has beneficial physiological effects such as improving insulin-stimulated whole-body glucose uptake, antioxidant capacity, and anti-inflammatory responses (5, 6). Additionally, Nieman et al., demonstrated the positive influence of fourteen days of quercetin (1,000 mg/day) exercise performance of 12-min time trial on the treadmill walking immediately after 60 min of moderate exercise preloads at 60% maximal oxygen uptake (V̇O_2max_) (7). McAnulty et al., evaluated the chronic quercetin effect on exercise-induced oxidative damage and inflammation, they recruited fourteen trained male cyclists who consumed 1,000 mg quercetin each day for 6 weeks before and during 3 days of cycling at 57% work maximum for 3 h (8). As compared to Nieman’s and McAnulty’s studies, we observe the participants in the present study performing 75% V̇O_2max_ cycling exercise and recorded the time to exhaustion for all subjects following a 3-h recovery period at 70% V̇O_2max_ exercise preload for 60 min under the shorter term of quercetin challenge (1,000 mg/day for 7 days). The questions of short-term quercetin and human exercise study need clarity regarding simulated cycling exercise challenge and post-exercise recovery during multiple races for cyclists in a one-day competition. However, some papers showing the ergogenic properties of quercetin were contrary (9–12). No effect of acute quercetin supplementation (2,000 mg) was found on 15 min cycling time trial following 30 min cycling exercise at a 50% V̇O_2_ peak under environmental circumstances of 40°C in non-heat-acclimated male volunteers by Cheuvront (9). Short-term (14 days) and long-term (6 weeks) quercetin supplementation (1,000 mg/d) both showed no ergogenic effect on the non-invasive measure of muscle oxidative capacity, physical fitness in healthy males, and V̇O_2_ peak measurement (10, 11). Moreover, Pelletier indicated that quercetin is unlikely to be evidence ergogenic for aerobic-oriented exercises in trained and untrained individuals by meta-analysis study (12). Nevertheless, whether the supplementation of quercetin alone can also improve the antioxidant enzymes and eliminate the oxidative stress caused by exercise remains unclear. Yet, no published human data are available regarding the effect of quercetin on post-exercise observation on whole-body insulin-stimulated glucose uptake, and indicators of oxidant stress, inflammation, muscle damage is a favor to improve subsequent cycling time to exhaustion. Especially, we wanted to demonstrate the effect of short-term quercetin supplementation (1,000 mg per day for 7 days) on exercise-induced oxidant damage and inflammation levels under simulating cycling competitions after exercise preloading.

It has been shown that the rate at which energy is replenished post-exercise, as well as decreased oxidative stress, inflammation, and muscle damage, is important for subsequent endurance exercise performance (13). Thus, athletes commonly use antioxidant supplements to accelerate energy recovery and eliminate oxidative stress and inflammation induced by exercise. In the present study, we explored whether 7-day quercetin supplementation can affect the blood energy metabolism index [glucose, insulin, and non-esterified fatty acids (NEFA)], oxidative markers [total antioxidant capacity (TAC), superoxidase dismutase (SOD), and MDA], inflammation markers [interleukin 6 (IL-6) and tumor necrosis factor-α (TNF-α)], muscle damage markers [CK, myoglobin (MB), and high sensitivity C-reactive protein (hs-CRP)], and time-to-exhaustion during two consecutive high-intensity cycling exercise bouts.

## Materials and Methods

### Subjects

The sample size was calculated in the present study using G* power (3.1.9.4) software assuming an effect size of 1.07 as observed previously in the human study regarding the ergogenic property of exercise time to exhaustion (14, 15). Therefore, 12 healthy male participants, physically active students, were recruited for this study supposing that the alpha error and statistic power are set at 0.05 and 90% (16). On average, their age was 20.79 ± 0.53 years old, the height of 171.10 ± 2.52 cm, bodyweight of 66.64 ± 2.67 kg, body mass index (BMI) of 22.86 ± 0.56 kg/m^2^, and the V̇O_2max_ of 45.18 ± 1.74 mL/kg/min, respectively. Prior to the experimental period, all participants were asked to familiarize with the bicycle ergometer, and perform the V̇O_2max_ test. During the recruiting process, the participants who were excluded included those whose chronic medical history such as cardiovascular disease, diabetes, musculoskeletal, or neuromuscular and were unable to complete the V̇O_2max_ test under trouble respiration. All participants kept their dietary habits and avoid consuming food, supplements, and beverages not including antioxidant and anti-inflammation (e.g., coffee, tea, cola, chocolate, drugs, and nutritional products) during the experimental period. The experimental protocol was approved by the Institutional Review Board at the University of Taipei, Taipei, Taiwan (UT-IRB-2018-085). The testing protocol was thoroughly explained to the participants and informed consent forms were signed in order to participate. Participants were allowed to voluntarily remove themselves from the study at any time without reason.

### Experimental Design and Procedure

**FIGURE 1:**
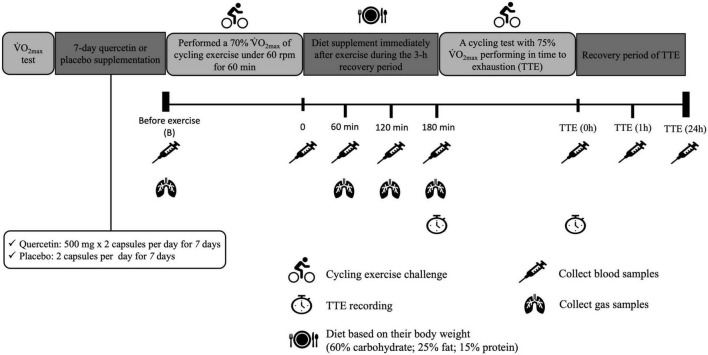
Experimental design and protocol.

Twelve healthy participants, physically active students, were recruited for the single-blind crossover study design. Participants were randomly assigned the placebo (P) or Quercetin (Q). The trial order for the 12 subjects was randomized so that six subjects with quercetin trial, while six subjects stared with the placebo. The crossover trial was repeated after completion of the first trial with the 14-day washout period, all subjects accept the implementation of the random allocation sequence occurring without the knowledge of which supplements were received by themselves using the identical appearance of the capsule with quercetin and placebo. The participants received placebo or quercetin for seven consecutive days with a 14-day washout period between the two trials, as shown in Figure 1, the participants’ V̇O_2max_ was measured, and the power for the cycle ergometer was calculated individually. During the trial, subjects were instructed to keep their diet habits, control the energy intake, receive the meals provided by investigators, and avoid strenuous exercise until the day of the experiment. Caffeine intake, smoking, ergogenic supplements, anti-inflammatory drugs, and food items including polyphenol and vitamins were not allowed. All participants received the light breakfast diet and the identical lunch and supper (total energy per meal of lunch/supper is 700.60 kcal, carbohydrate, 77.90 g; fat, 26.60 g; protein, 37.10 g) during the 24 h before trial. To achieve the simulating glycogen depletion was performed in the first cycling exercise challenge, participants fasted for 12-h before the day of the experiment. At 7 am on the next morning, participants reported to the lab and their height and body weight were measured, followed by a cycling challenge at 70% of V̇O_2max_ for 60 min while maintaining 60 rpm (17). Immediately after the first exercise challenge, participants consumed the light diet contained 60% carbohydrate with 1,000 mg quercetin/placebo (60% carbohydrate, 24.12 ± 0.20 g; 25% fat, 16.82 ± 0.21g; 15% protein, 16.12 ± 0.20 g; 304.12 ± 1.20 kcal). Approximately 3 h (post-exercise recovery) later, participants performed a cycling test at 75% of V̇O_2max_ until exhaustion. Time-to-exhaustion was recorded. Blood and gas samples were collected before and after the first cycling bout, during post-exercise recovery, and after exhaustion.

### Maximal Oxygen Uptake Test

Participants performed the V̇O_2max_ test on a cycle ergometer (Monark, Varberg, Sweden) while each breath was assessed with gas analyzers (Cortex Biophysik, Non-nenstrasse, Leipzing, Germany). The participants maintained 60 RPM’s throughout the test. The primary workload was at 0.5 kg for 4 min and then increased by 0.5 kg every 2-min until exhaustion. The determination of V̇O_2max_ was based on three criteria: (1) Respiratory exchange ratio (RER) > 1.10; (2) V̇O_2max_ variance < 2 mL/kg/min; and (3) target heart rate reaches a theoretical maximum value at “220-age” (18). Accordingly, the value obtained from the Y-axis was oxygen consumption (ml/kg/min); the value obtained from the X-axis was the workload that corresponded to the oxygen uptake. V̇O_2max_ (100%) was determined when the curve reached the plateau. The value multiplied by 0.70 was defined as 70% V̇O_2max_ as the intensity for the cycling challenge; multiplied by 0.75 was defined as 75% V̇O_2max_ as the intensity for the subsequent time-to-exhaustion test, and the corresponding value from X-axis was the workload used during the formal experiments (19).

### Quercetin and Placebo Supplement

The participants received placebo or quercetin for seven consecutive days with a 14-day washout period between the two trials. During seven consecutive days, the participants ingested either two quercetin capsules or two placebo capsules after breakfast at 8 am in the lab. On the exercise challenge experimental morning, the participants ingested either two quercetin capsules or two placebo capsules immediately after the first exercise challenge. Each quercetin capsule (GNC Holdings Inc., United States) was 500 mg while each placebo capsule was 500 mg of cellulose. Thus, the participants received 1,000 mg quercetin or cellulose per day. This dosage of quercetin was within safe limits for human intake (20).

### Blood Sample Collection and Analysis

Blood samples were collected before exercise, 1*^st^* exercise recovery period, and 2nd post-exercise 0, 1, and 24 h. All blood samples were tested for the exercise-induced status of oxygen stress, inflammation, and muscle damage. Blood samples were collected and centrifuged at 1,000 *g* for 10 min. Afterward, the supernatant was collected and placed in a freezer at –20°C. The supernatant was used to measure the following: insulin, NEFA, TAC, SOD, MDA, IL-6, TNF-α, CK, MB, and hs-CRP. Blood glucose concentration was determined by an automated glucose analyzer (YSI Life Sciences). Plasma insulin levels were determined using a commercial kit (Roche Diagnostics, Mannheim, Germany). Streptavidin microparticle interacted with anti-insulin AB-biotin and anti-insulin AB-Ru (bpy) 32+ to emit chemiluminescence (Roche Elecsys 1010/2010, Roche Diagnostics; MODULAR ANALYTICS E170, Roche Diagnostics) for analyzing the insulin levels and calculation of insulin sensitivity index (ISI) (21). Plasma NEFA was measured using a commercial kit (Wako, Neuss, Germany) in combination with an automated biochemical analyzer (Hitachi Science Systems, Ltd., Lbaranki, Japan). TAC, MDA, SOD, IL-6, and TNF-α can be used to determine the status of oxidative stress and inflammation. TAC, MDA (Sigma, St. Louis, MO, United States), and SOD (Cayman Chemical Company, ANN Arbor, MI, United States) were measured using commercial kits (Tecan GENios, A -5082, Austria). The levels of TAC, MDA, and SOD were determined by absorbance at 734, 532, and 570 nm wavelength using the standard curve. Cytokine IL-6 (Bio-Legend Inc., San Diego, CA, United States) and TNF-α (Bio-Legend Inc., San Diego, CA, United States) were measured using a commercially available reader (Tecan GENios, A-5082, Austria) to read the absorbance at 450 and 570 nm wavelengths to calculate concentrations in the serum samples. Additionally, CK, MB, and hs-CRP served as the biomarker for muscle damage. CK (Cayman Chemical Company, ANN Arbor, MI, United States), MB (Wuhan Fine Biotech Co., Ltd., Hubei, China), and hs-CRP (Denka Seiken Co., Ltd., Tokyo, Japan) were measured using commercial kits. The concentration was determined using the reader (Tecan GENios, A-5082, Austria) to obtain absorbance at 450 and 570 nm wavelengths as mentioned in the previous section.

### Gas Sample Collection and Analysis

Gas samples were collected via the gas analyzer every 60 min for 3 hours (for example, 60, 120, and 180 min) to determine RER during the post-exercise recovery period after subjects were given a carbohydrate-rich diet. RER was calculated based on the amount of CO_2_ generated/VO_2_.

### Statistics

Values were presented as mean ± standard error (mean ± SE). SPSS software was used for statistical analysis. Paired t-test was used to analyze time-to-exhaustion. Repeated measure two-way ANOVA (trial × time) was used to determine the intra- and inter- difference at different time points and between the two groups with power set at 80%. If significant interaction was observed within and between groups, a simple main effects analysis was conducted. Fisher’s least significant difference (LSD) was used for *post hoc* analysis. The α value was set at *p* < 0.05.

## Results

### Quercetin Enhanced the Subsequence High-Intensity Cycling Performance to Exhaustion After 3-h Post-exercise Recovery of the First Exercise Challenge

**FIGURE 2:**
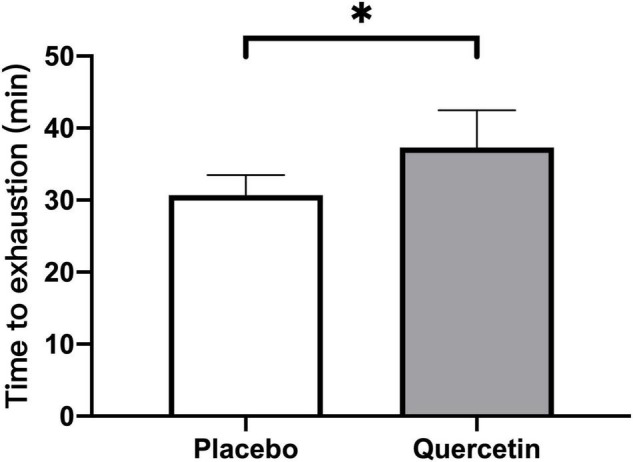
Mean values data of all participants for the time-to-exhaustion tests of the second cycling exercise with 75% VO_2max_. *Significant difference between quercetin and placebo (*p* < 0.05). Values are expressed as mean ± SE, *N* = 12.

**FIGURE 3:**
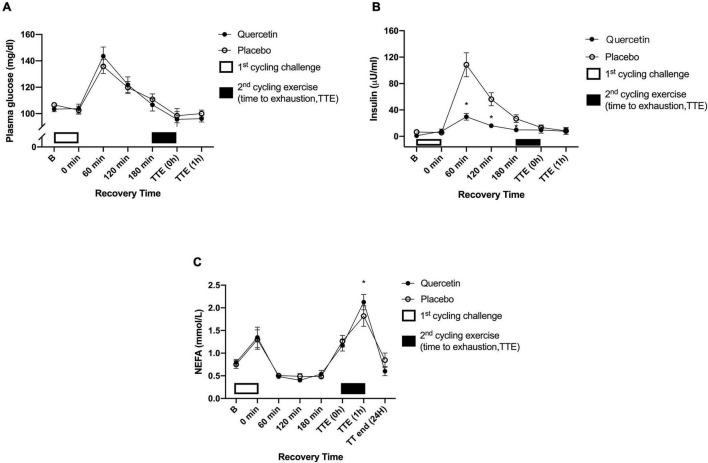
Blood glucose **(A)**, plasm insulin **(B)**, and plasma non-esterified fatty acids (NEFA) **(C)** concentrations in (-•-) quercetin and (-◦-) placebo trials. B represents before the first cycling challenge. TTE (0,1 h,24 h): represents immediately, 1-h, and 24-h after the second cycling exercise, respectively. *Significant difference between quercetin and placebo (*p* < 0.05). Values are expressed as mean ± SE, *N* = 12.

**FIGURE 4:**
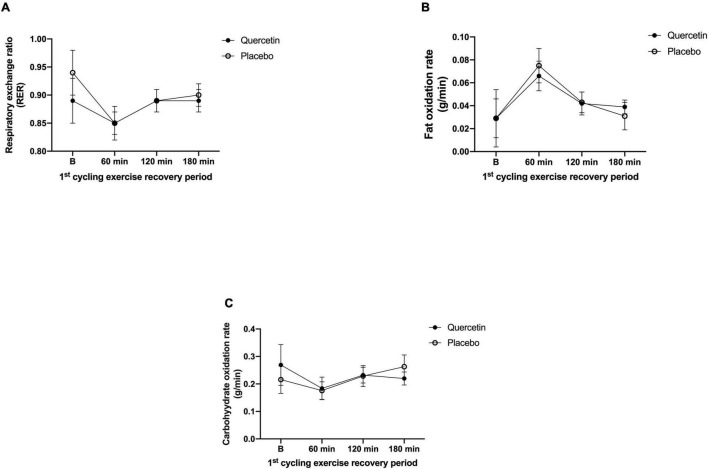
Respiratory exchange rate **(A)**, fat oxidation rate **(B)**, and carbohydrate oxidation rate **(C)** in (-•-) and quercetin trials (-◦-) placebo. B represents before the first cycling challenge; *Significant difference between quercetin and placebo (*p* < 0.05). Values are expressed as mean ± SE, *N* = 12.

We found that seven days of quercetin significantly enhanced the second cycling exercise performance to exhaustion (Quercetin: 37.29 ± 5.20 min; Placebo: 30.72 ± 2.16 min, *p* < 0.05) (Figure 2). Significantly lower insulin responses were found in quercetin than those of placebo at 60, 120, and 180-min after the first cycling exercise challenge (Figure 3B, *p* < 0.05). However, there was no significant difference in glucose and NEFA (Figures 3A,C) during the experimental procedures. No significant difference in whole-body carbohydrate and fat oxidation were found between the two trials calculated by gaseous samples during the first cycling exercise recovery period (Figure 4, *p* > 0.05).

### Quercetin Enhanced Antioxidant Capacity and Resulted in Decreased Oxidative Stress After the Cycling Exercise Challenge

**FIGURE 5:**
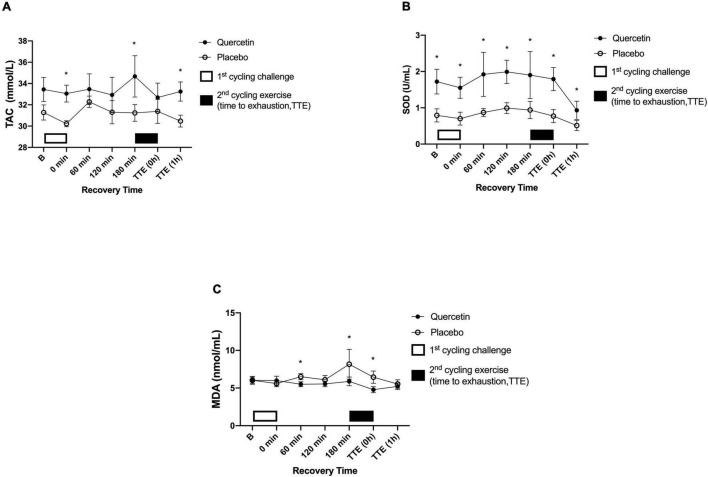
Serum total antioxidant capacity (TAC) **(A)**, superoxidase dismutase (SOD) **(B)**, and malondialdehyde (MDA) **(C)** concentrations in (-◦-) placebo and (-•-) quercetin trials. B represents before the first cycling challenge. TTE (0h,1h): represents immediately, and 1-h after the second cycling exercise, respectively. *Significant difference between quercetin and placebo (*p* < 0.05). Values are expressed as mean ± SE, *N* = 12.

Total antioxidant capacity activity was enhanced by quercetin, evidenced by the response after the cycling exercise challenge (Figure 5A, *p* < 0.05). Similarly, the SOD concentrations were significantly higher in the quercetin than those of placebo (Figure 5B, *p* < 0.05). In addition, we measured oxidative stress MDA markers, which were significantly lower at 60, and 180-min during the first exercise recovery period and at the end of the second cycling exercise performance to exhaustion (Figure 5C, *p* < 0.05).

### Quercetin Attenuated IL-6 and CK Levels Induced by the High-Intensity Cycling Exercise to Exhaustion After 3 h Post-eercise Recovery of 75% VO_2max_ Exercise Challenge

**FIGURE 6:**
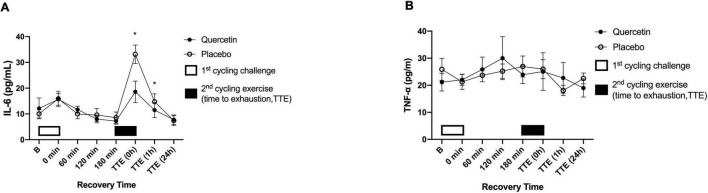
Serum interleukin-6 (IL-6) **(A)** and tumor necrosis factor-α (TNF-α) **(B)** concentrations in (-◦-) placebo and (-•-) quercetin trials. B represents before the first cycling challenge. TTE (0 h, 1 h, 24 h): represents immediately, 1-h, and 24-h after the second cycling exercise, respectively. *Significant difference between quercetin and placebo (*p* < 0.05). Values are expressed as mean ± SE, *N* = 12.

**FIGURE 7:**
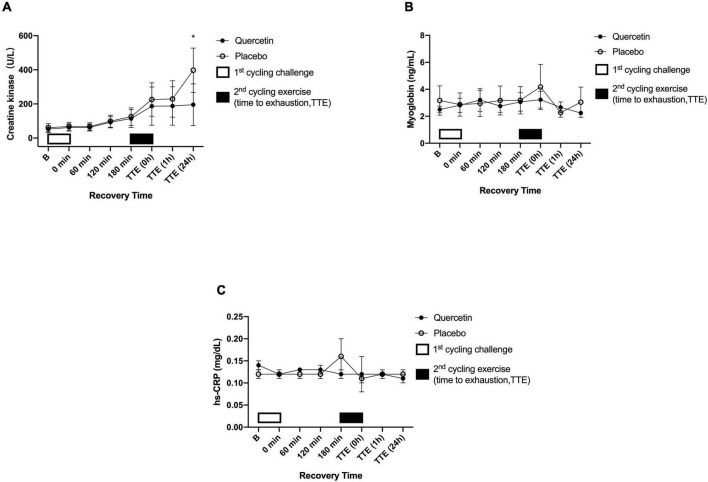
Serum creatine kinase (CK) **(A)**, myoglobin **(B)**, and hs-CRP **(C)** concentrations in (-◦-) placebo and (-•-) quercetin trials. B represents before the first cycling challenge. TTE (0 h, 1 h, 24 h): represents immediately, 1-h, and 24-h after the second cycling exercise, respectively. *Significant difference between quercetin and placebo (*p* < 0.05). Values are expressed as mean ± SE, *N* = 12.

The effect of quercetin on circulating response regarding inflammation and muscle damage indicators was measured before and after two bouts of cycling exercise challenge. Figures 6A, 7A showed the significant response of pro-inflammatory markers IL-6 and muscle damage indicators CK after Quercetin supplementation (*p* < 0.05). In this human study, IL-6 concentrations were significantly lower immediately, and 1-h after the second cycling exercise (Figure 6A, *p* < 0.05). The peak serum CK responded at 24-h after the second cycling challenge (Figure 7A). Identically, the concentrations of the serum CK were significantly lower at 24-h in the quercetin trial compared to the placebo trial (Figure 7A, *p* < 0.05). However, there were no differences in TNF-α (Figure 6B), MB (Figure 7B), and hs-CRP (Figure 7C) between the two trials. Therefore, we speculate that oral quercetin supplementation tends to attenuate IL-6 and CK concentrations after high-intensity cycling exhaustion challenges but no other inflammation and muscle damage indicators.

## Discussion

Quercetin is a polyphenolic flavonoid with antioxidant and anti-inflammatory properties (22–25). In the present human study, we hypothesized that 7-days of oral quercetin supplementation can boost antioxidant and anti-inflammatory responses as well as mitigate oxidative stress and inflammation caused by high-intensity cycling for 60 min. Furthermore, we hypothesized that supplementation would increase time to exhaustion during the second high-intensity cycling challenge. The major finding in the present study was significantly increased time-to-exhaustion during the second cycling challenge after a short-term (3 h) break following the first cycling bout (Figure 2). The compatible papers showing the ergogenic effect of quercetin are reported (7, 22, 23, 26). Nevertheless, some comparisons were made where the quercetin supplement in four published papers indicating the ergogenic properties of quercetin on exercise performance was contrary to the present study (9–12). The short-term model of quercetin supplementation in the present study is not the same as the acute condition as Cheuvront’s study and the blood sampling about oxidant stress, inflammation, and muscle damage indicators in the present study are different from the non-invasive measurement of muscle oxidative capacity, physical fitness in Cureton or Bigelman studies. Nonetheless, we conservatively inferred that 1,000 mg/day of quercetin supplementation for a week improved a time trial performance following a moderate aerobic exercise, evidenced on post-exercise blood samples regarding increased systemic insulin-stimulated glucose uptake, antioxidant capacity, and decreased IL-6, CK levels.

As shown in Figure 3B, insulin levels by quercetin were significantly lower than those of placebo during the first post-exercise recovery period. The calculation of area under the curve for glucose/insulin and insulin sensitivity index (ISI) are shown (IACU: Quercetin:3248.88 ± 1643.56 min × μU/ml; Placebo:13085.78 ± 6178.03 min × μU/ml, *p* < 0.05; GACU: Quercetin:671.50 ± 13.63 min × mg/ml; Placebo: 670.70 ± 17.51 min × mg/dl, *p* > 0.05; ISI: Quercetin: 24.85 ± 14.7; Placebo: 4.95 ± 1.32, *p* < 0.05). Presumably, these data led to increased muscle glycogen levels and endurance performance during subsequent exercises (27). In previous studies, Cheng et al. performed a single-blind, randomized, crossover study that involved post-exercise supplementation of hydroxycitric acid or placebo for a group of male subjects. The supplement significantly accelerated the rate of glycogen synthesis twofold in exercised human skeletal muscle compared to placebo; this increase occurred in parallel with increased whole-body insulin-stimulated glucose uptake, evidenced by reduced post-meal insulin response to the same carbohydrate meal challenge (28). Therefore, the synthesis of glycogen from glucose was promoted which is especially meaningful to energy replenishment during post-exercise recovery (13, 29). Guo et al. study showed that quercetin can positively regulate insulin receptor substrate 1 (IRS-1) serine/tyrosine phosphorylation and regulate insulin signaling through the downstream protein kinase B (Akt)/endothelial nitric oxide synthase (eNOS)-phosphoinositide 3-kinase (PI3K) pathway (30). Unfortunately, the present study did not perform the muscle biopsy to assess muscle glycogen results, and systemic fat oxidation utilization was not significantly enhanced by quercetin during post-exercise recovery (Figure 4). Therefore, we suggest that the higher insulin-stimulated glucose uptake found in this study enhanced replenishment function which would be conducive to the rapid restoration of muscle glycogen. This would then have a synergistic effect to improve time-to-exhaustion during exercise.

Following 7-days of quercetin supplementation antioxidant enzymes, TAC and SOD were changed significantly in the present study. Quercetin has been shown the most potent scavenger of ROS (31). The anti-oxidative capacities of quercetin are due to the presence of two antioxidant pharmacophores within the molecule that have the optimal configuration for free radical scavenging, i.e., the catechol group in the B ring and the OH group at position 3 of the AC ring (32). In the present study, the SOD activity and TAC by quercetin were significantly higher than those of placebo during the experimental period in parallel with significantly decreased levels of MDA, a plasma lipid peroxidation product caused by exercise (Figures 5A–C). TAC and SOD are considered the first line of defense against ROS, catalyzing the decomposition of superoxide anions into hydrogen peroxide (33). These findings appear to be in line with the supplement itself. Quercetin, a natural polyphenolic flavonoid, has been shown to provide a combination of antioxidant and anti-inflammatory properties in both animal and human studies (5, 6). The antioxidant capacity of quercetin has been attributed to its chemical structure, especially the presence and position of the catechol group in the B ring and the hydroxyl (–OH) group, which regulate the redox mechanism and enhance the ability of free radical scavenging (34). It has been reported that high oxidative stress in the human body significantly postpones or reduces the mitochondrial synthesis in skeletal muscle (35) and affects the rate of cellular adenosine triphosphate (ATP) synthesis (36). Previous human studies showed that mitochondrial synthesis and ATP production play important physiological roles to improve human exercise performance (7). Additionally, Paschalis et al. reported that antioxidant supplements significantly decrease oxidative stress as measured by F2-isoprostanes and protein carbonyls, which effectively improved the aerobic performance (V̇O_2max_ test), anaerobic power test (Wingate), and 5-min time trial test (37). Almeida et al. reported that quercetins absorption was variated by their gut microbiota in the small intestine and low bioavailability in humans (38). Diksha et al., study was to investigate the absorption of quercetin in 18 healthy human subjects administered. The C_max_ of quercetin was highest achieved within 3.3 h (39). The present study was designed to perform multiple exercises over a long period of time, consisting of an initial 60-min 70% V̇O_2max_ test followed by a 3 h post-exercise recovery period, and finishing with high-intensity cycling exercise to exhaustion. Similar to Paschalis’ human study, the increased antioxidant enzymatic activity, coinciding with reduced oxidative stress, supports quercetin supplementation for improving high-intensity exercise performance.

Physiological inflammation levels and increased muscle damage are related to both the muscles exercised and for how long the muscles were actively engaged. For instance, whole-body muscle movement has been reported to significantly increase the concentration of IL-6, TNF-α, CK, MB, and hs-CRP (40–42). A previous study from our lab indicated that IL-6 levels significantly increased in response to a cycling challenge at 80% V̇O_2max_ (43). The same exercise was applied in the present and significantly increased levels of IL-6 after exhaustion for both the quercetin and placebo groups (Figure 6A), indicating that treatment with exercise at 75% V̇O_2max_ could induce pro-inflammatory marker IL-6 levels in the human body. Quercetin has been shown the property of anti-inflammation via the inhibition of activation of the transcript factors such as nuclear factor-κB (NF-κB) and activator protein (AP-1) (44, 45). Consequently, the property of quercetin on scavenging ROS would not only prevent the occurrence of oxidative stress but also help mitigate inflammation. Indeed, it has already been shown that quercetin can inhibit pro-inflammation via modulation of NF-κB in human peripheral blood mononuclear cells (46). The present study monitored physiological reactions throughout the trial in participants treated with quercetin (1,000 mg/day) for 7 days. The IL-6 levels caused by exercises were significantly reduced by 7-day quercetin supplementation (Figure 6A, *p* < 0.05). Together, we were able to infer that 7-day quercetin supplementation was capable of inhibiting the muscle pro-inflammatory cytokine IL-6 concentration induced by high-intensity exercise to exhaustion, suggesting that quercetin supplementation can assert positive physiological effects. In addition, blood CK and MB are sensitive to muscle damage and serve as biomarkers for such damage. Creatine kinase participates in the reversible reaction that catalyzes the phosphorylation of creatine, ultimately forming new ATP, which occurs primarily in skeletal muscle (47), the effect of quercetin on CK response involving AMP activated protein kinase (AMPK) itself a regulation in the heart muscle of rats (48, 49). In the present study, we speculated the conservation of the adenine nucleotide pools in the skeletal muscle may be a supplementary advantage of quercetin regarding ergogenic strategy for athletes. The present study investigated the positive effects of quercetin supplementation for 7 days on muscle damage biomarkers and showed that CK was significantly lower than placebo at 24-hr after the exercise-to-exhaustion challenge (Figure 7A, *p* < 0.05), not in MB, and hs-CRP (Figures 7B,C, *p* > 0.05). The authors wondered that unsuitable blood sample timing to correctly measure MB, and hs-CRP was likely the reason for our inability to detect changes. Cunniffe et al. showed that serum cortisol and IL-6 levels increased immediately after an International Rugby League game. While CK and the CRP index reached their peaks 14 and 38 hours after exercise respectively, perhaps revealing the true pattern of these physiological biomarkers in the acute phase post-exercise (50), suggesting that the time at which blood samples are obtained is crucial. Nevertheless, the last time-point of blood sampling in the present human study was 24 h after exhaustion, which may not be appropriate to observe the course of change among inflammation and muscle damage markers such as MB, and hs-CRP.

In conclusion, this study examined the effects of quercetin (1,000 mg/day for 7 days) supplementation on insulin sensitivity, antioxidant capacity, muscle damage, and exercise performance in human subjects. Quercetin supplementation was found to improve whole-body insulin-stimulated glucose uptake and antioxidant capacity, which attenuated exercise-induced oxygen stress during two consecutive high-intensity cycling bouts. Following a 3-h recovery period from the first high-intensity exercise session, quercetin improved time to exhaustion for the subsequent cycling test. Actually, there were limitations in the present study, including without performing a muscle biopsy, due to the COVID-19 pandemic, the muscle biopsy could not be performed on the exercised subjects to obtain muscle glycogen data, therefore, we speculated the effect of muscle glycogen use on cycling time to exhaustion only based on insulin data without to outline the possible underlying mechanisms for a relationship between muscle glycogen and cycling time to exhaustion. Of course, we also were lack of records about physical activity and dietary diaries during the experimental periods for all participants. This useful information could elucidate the exclusive benefit effect of quercetin in the ergogenic property on exercise competition. However, the novel findings and new knowledge the present study provided was quercetin supplementation enhanced the post-exercise whole-body insulin-stimulated glucose uptake and antioxidant capacity, in parallel with a decrease in exercise-induced MDA, IL-6, and CK levels throughout the post-exercise period. The beneficial effects of quercetin are likely due to its glucogenic, antioxidant, and anti-inflammatory properties. Thus, short-term quercetin supplementation may be considered an effective ergogenic aid for enhancing high-intensity cycling performance among young adults.

## Data Availability Statement

The raw data supporting the conclusions of this article will be made available by the authors, without undue reservation.

## Ethics Statement

The studies involving human participants were reviewed and approved by Institutional Review Board at the University of Taipei, Taipei, Taiwan (UT-IRB-2018-085). The patients/participants provided their written informed consent to participate in this study.

## Author Contributions

I-SC designed the experiments. J-PT, S-FL, and H-CH carried out the laboratory experiments. J-PT and C-LH contributed reagents, materials, and analysis platforms. S-FL, C-LH, and I-SC analyzed the data. JB helped with the polishing of English writing. J-PT and I-SC interpreted the results, prepared the figures, and wrote and revised the manuscript. All authors are qualified and approved this final submitted version of this study.

## Conflict of Interest

The authors declare that the research was conducted in the absence of any commercial or financial relationships that could be construed as a potential conflict of interest. The handling editor MK declared a past collaboration with the authors S-FL, I-SC, and J-PT at the time of review.

## Publisher’s Note

All claims expressed in this article are solely those of the authors and do not necessarily represent those of their affiliated organizations, or those of the publisher, the editors and the reviewers. Any product that may be evaluated in this article, or claim that may be made by its manufacturer, is not guaranteed or endorsed by the publisher.
